# Synthesis of β-Cyclodextrin@gold Nanoparticles and Its Application on Colorimetric Assays for Ascorbic Acid and *Salmonella* Based on Peroxidase-like Activities

**DOI:** 10.3390/bios14040169

**Published:** 2024-03-31

**Authors:** Xinyi Fan, Yuexin Bao, Yanhong Chen, Xiaohong Wang, Stephen L. W. On, Jia Wang

**Affiliations:** 1 College of Food Science and Technology, Huazhong Agricultural University, Wuhan 430070, China; skfzmaildc@163.com (X.F.); baoyuexin@webmail.hzau.edu.cn (Y.B.); 18434763324@163.com (Y.C.); wxh@mail.hzau.edu.cn (X.W.); 2 Key Laboratory of Environment Correlative Dietology, Huazhong Agricultural University, Wuhan 430070, China; 3 Department of Wine, Food and Molecular Biosciences, Faculty of Agriculture and Life Sciences, Lincoln University, Lincoln 7647, New Zealand; stephen.on@lincoln.ac.nz

**Keywords:** gold nanoparticles, β-cyclodextrin, peroxidase-like behavior, ascorbic acid, *Salmonella* Typhimurum

## Abstract

The peroxidase-like behaviors of gold nanoparticles (AuNPs) have the potential to the development of rapid and sensitive colorimetric assays for specific food ingredients and contaminants. Here, using NaBH_4_ as a reducing agent, AuNPs with a supramolecular macrocyclic compound β-cyclodextrin (β-CD) capped were synthesized under alkaline conditions. Monodispersal of β-CD@AuNPs possessed a reduction in diameter size and performed great peroxidase-like activities toward both substrates, H_2_O_2_ and TMB. In the presence of H_2_O_2_, the color change of TMB oxidization to oxTMB was well-achieved using β-CD@AuNPs as the catalyst, which was further employed to develop colorimetric assays for ascorbic acid, with a limit of detection as low as 0.2 μM in ddH_2_O. With the help of the host-guest interaction between β-CD and adamantane, AuNPs conjugated with nanobodies to exhibit peroxidase-like activities and specific recognition against *Salmonella* Typhimurium simultaneously. Based on this bifunctional bioprobe, a selective and sensitive one-step colorimetric assay for *S*. Typhimurium was developed with a linear detection from 8.3 × 10^4^ to 2.6 × 10^8^ CFU/mL and can be provided to spiked lettuce with acceptable recoveries of 97.31% to 103.29%. The results demonstrated that the excellent peroxidase-like behaviors of β-CD@AuNPs can be applied to develop a colorimetric sensing platform in the food industry.

## 1. Introduction

Gold nanoparticle (AuNPs)-based colorimetric assays are of great interest for the identification of chemical compounds and biological contamination in the fields of food and environmental monitoring and biomedical applications [1]. AuNPs, normally with a diameter of 1~100 nm, possess localized surface plasmon resonance (LSPR) character, excellent conductivity and specific molecular recognition via their surface electrostatic adsorption or labeled with desirable antibodies or aptamers [2,3]. Generally, bare AuNPs with/without labeled undergo aggregation to give rise to a red-shift of LSPR absorbance with a significant color change from red to blue, which represents the signal transducing for colorimetric assay development [4]. 

Since peroxidase-like properties, as first demonstrated by Fe_3_O_4_ NPs in 2007 [5], attentions on the catalytic activities of AuNPs has been attracted to be used to develop colorimetric methods. The nanoparticles possessing catalytical activities as conventional enzymes to catalyze the substrates are also referred to as nanozymes [6]. Take the peroxidase-like properties as an example: 3,3′,5,5′-tetramethylbenzidine (TMB), used as substrates, can be oxidized to oxTMB with a change in colorimetric signals, which can be identified easily by naked eyes or simple colorimetric devices. The catalytic performances of nanoparticles are influenced by the particle size, shape, surface chemical groups and synthetic methods [7]. AuNPs with a typical size below 2 nm (always named gold nanoclusters) have shown good catalytic activities, probably due to the increase in the specific surface area [8]. Surface functionalization of AuNPs is an effective strategy to prevent particle aggregation, which commonly occurs for AuNPs storage, and can enhance their catalytic properties [9]. The modified molecules on the AuNPs surface can also regulate their catalytic efficiencies and enhance their physical and chemical properties [10], such as cyclodextrins (CDs). The CDs possess a hydrophobic interior with a hydrophilic exterior and can incorporate hydrophobic molecules and ions to form unique host–guest inclusion compounds for application in biomedical and food industries [11,12]. Obtained from the degradation of starch in the presence of cyclodextrin glycosyltransferase, the most common CDs are the α-, β- and γ-forms, including six, seven and eight glucose units, with the different cavity volumes of 0.174, 0.262 and 0.427 nm^3^, respectively [13]. Therefore, the host–guest chemistry of CDs can endow AuNPs with unique properties, which can be used for particle-aggregation-based colorimetric biosensor development [14,15,16,17] and catalytic activity enhancements [18].

In order to fix CDs on the surfaces of AuNPs, the Au-O bond and Au-S bond are the two main groups to form covalent bonds with CDs. Thus, CDs or their thiolated form can act as both reducing and stabilizing agents to react directly with chloroauric acid under the condition of high temperatures [18,19], representing catalytically glucose oxidization to gluconic acid [18], hydrolysis of carbonate, peroxidase-mimicking and esterase-mimicking activities [20]. However, the stringent conditions for synthesis and particle size ranging from 20 to 30 nm might affect their catalytical activities and further their applications. Introducing sodium borohydride or other reducing reagents can be an alternative approach to synthesizing CD@AuNPs under mild conditions and with a decrease in particle diameter sizes. Thus, in this study, using CDs as a stabilizer and sodium borohydride as a reducing agent, CD@AuNPs were synthesized at ambient temperature, forming a diameter size of approximately 5 nm within 30 min. Then, the peroxidase-like activities of CD@AuNPs were exploited to develop colorimetric assays to determine ascorbic acid (AA) and *Salmonella* Typhimurium for portable detection.

## 2. Materials and Methods

### 2.1. Chemicals and Reagents

Hydrogen tetrachloroaurate (III) trihydrate (99.9%) was purchased from Sigma-Aldrich (Milwaukee, WI, USA). α-cyclodextrin (α-CD), β-cyclodextrin (β-CD) and γ-cyclodextrin (γ-CD) were acquired from Aladdin (Shanghai, China). Sodium borohydride (NaBH_4_), 1-adamantane carboxylic acid, 3,3′,5,5′-tetramethylbenzidine (TMB), skim milk, 1-Ethyl-(3-dimethylaminopropyl)carbodiimide (EDC) and N-hydroxysuccinimide sodium (NHS) were purchased from Macklin (Shanghai, China). All chemicals were of analytical grade. 

### 2.2. Synthesis of CD@AuNPs

Glassware for gold nanoparticle synthesis was immersed in fresh Aqua Regia (HNO_3_/HCl = 1:3, *v*/*v*) and rinsed with Milli-Q ultrapure water before use. The CD@AuNPs were prepared using the method of Song et al. [21] with some modifications. 0.4 mM of HAuCl_4_ solution (50 mL) was prepared with 0.2 mol/L of K_2_CO_3_ for pH adjustment. Then, 12.5 mM of fresh NaBH_4_ was added to the conical flask in the presence of 0.5 mM of CD in the HAuCl_4_ solution. The reaction remained at ambient temperature for a further 30 min after changing from light yellow to red. Solutions were filtered (0.45 μm) and stored in a refrigerator at 4 °C. The conditions for CD@AuNPs synthesis were optimized by α-CD, β-CD and γ-CD replacement, pH values and the molar ratios of β-CD:HAuCl_4_ and NaBH_4_:HAuCl_4_. The absence of CD was employed in the same procedures. The optimized conditions were determined by the evaluation of the size and dispersivity of CD@AuNPs particles, followed by being characterized using UV-vis spectroscopy, transmission electron microscopy (TEM) micrographs, Fourier transform infrared spectroscopy (FTIR) and ^1^H NMR spectrum. 

### 2.3. Simulation of Peroxidase Activities by β-CD@AuNPs

A total of 500 μL of citric acid buffer (0.01 M, pH 3.5), 25 μL of TMB solution (5 mM) and 15 μL of H_2_O_2_ (30%) were freshly mixed and then added into a tube with/without 100 μL of β-CD@AuNPs. After incubation at 37 °C for 30 min, the absorption spectra of the solution were scanned, ranging from 500 nm to 800 nm, using a UV-Vis spectrophotometer (UV-2450, Shimadzu, Japan). The pH value (3.0, 3.5, 4.0, 4.5, 5.0, 5.5, 6.0 and 6.5), incubation temperature (25, 37, 42, 55 and 65 °C) and time (0, 5, 10, 15, 20, 30, 40, 50 and 60 min) were used separately to evaluate their effects to the simulation of peroxidase activities in the synthesis of β-CD@AuNPs.

Kinetic analysis of β-CD@AuNPs was performed, and the initial rate was assessed within the first 5 min by fixing one of the concentrations of H_2_O_2_ and TMB and varying the other. The final concentration of H_2_O_2_ was fixed to 40 mM and the fixed TMB was set to 0.6 mM. The kinetic parameters of initial reaction rate (V) were calculated as the equation $V=\frac{Vmax\;\times \;[S]}{Km\;+\;[S]}$, with Km for Mi’s constant, [S] for the substrate concentration and Vmax for maximum reaction rate. 

### 2.4. Colorimetric Detection for Ascorbic Acid

The AA solution was freshly prepared and diluted with ddH_2_O. The mixture of 0.05 M citric acid buffer (pH 3.5), 5 mM TMB and H_2_O_2_ solution was added to β-CD@AuNPs (100 µL) in a 96-well microplate and incubated for 30 min at 37 °C. Then, a series concentration of AA (0.5 µM–8 µM, 75 µL) was added for another 20 min incubation. The optical density at 652 nm was measured using a microplate photometer. The limit of detection of this assay was determined as the average OD450 nm of the blank samples + 3.3SD. Instead of AA, ddH_2_O was set as a negative control (A_0_). High concentrations of NaCl (6 mM), KCl (6 mM), CaCl_2_ (6 mM), glucose (2.5 mM), maltose (2.5 mM), sucrose (2.5 mM), fructose (2.5 mM), histidine (6 mM), valine (2.5 mM), glycine (2.5 mM), L-cystine (0.2 mM) and BSA (0.2 mM) were added to evaluate the specificity of the colorimetric assay. 

### 2.5. Colorimetric Detection for S. Typhimurium

#### 2.5.1. Culture of Bacteria

All the bacterial strains including competent cells *E. coli* Top 10f’, *E. coli* BL21(DE3), *S*. Paratyphi B CMCC 50094, *S*. Enteritidis ATCC 13076, *Vibrio parahaemolyticus* ATCC 17802, *E*. *coli* ATCC 12900, *Staphylococcus aureus* ATCC 25923 and *Pseudomonas aeruginosa* CMCC 10104 were preserved in our laboratory. All pathogenic bacterial strains were revived on Luria-Bertani agar plates before use.

#### 2.5.2. Preparation of Nanobodies and Its Conjugation with Adamantane (Ada)

Nanobody against *S*. Typhimurium (VHH-9-28) was obtained through biopanning from an immunized VHH library displayed on phage particles in our laboratory (data not published). Its expression and purification were carried out as follows: The recombinant plasmid pComb3X-VHH encoding the VHH-9-28 gene was chemically transformed into *E. coli* Top10f’ competent cells. Confirmed by sequence analysis, a single positive colony was used for nanobody expression, with 1 mM of IPTG (final concentration) induction and cell lysis by B-Per solution (4 mL/g pellets). The soluble nanobody expression in periplasm was collected and used for purification by nickel-resin affinity chromatography, with its purity analyzed using SDS-PAGE (concentrated gel 4%, separation gel 12%). The purified nanobody was dialyzed for 24 h before conjugating with adamantane. The preparation of ada-VHH was carried out according to the reported method [22] with slight modifications. Briefly, qual molar of EDC and NHS were prepared and successively added to 2 mL of adamantane carboxylic acid solution. Subsequently, 700 μL of VHH (0.15 mg/mL) was gently added, and the reaction was carried out overnight at 4 °C. The excess EDC and NHS were dialyzed for 24 h in PB buffer (0.01 mM).

#### 2.5.3. Colorimetric Assays for *S.* Typhimurium Based on VHH-AuNPs

The conjugates VHH-AuNPs were constructed by mixing ada-VHH and β-CD@AuNPs at a molar ratio of 1:1 and stirring overnight in the dark. After dialysis overnight, VHH-AuNPs were characterized by UV-Vis absorption spectroscopy with a wavelength of 200~800 nm. A single clone of *S*. Typhimurium ATCC 14028 was selected on the XLD plate and inoculated into a 3 mL LB medium to shake until achieving a concentration of 10^8^ CFU/mL. Then, the bacteria (100 μL/well), boiled for 20 min for inactivation, were used to coat on a microplate overnight. By washing them with 0.5% Tween-20 in distilled H_2_O, the wells were blocked with 250 μL of skim milk and incubated for 1 h at 25 °C. Subsequently, VHH-AuNPs (100 μL/well) were added and incubated to bind with the target bacteria. After removing the unbound VHH-AuNPs, the substrate (100 μL pH 3.5 citric acid buffer, 25 μL TMB, 15 μL H_2_O_2_) was added to the wells and reacted for 30 min. Followed by termination with 2 M H_2_SO_4_, the OD450nm was determined using a microplate reader (Molecular Devices, Sunnyvale, CA, USA). Distilled H_2_O, substituted for bacteria, was used as the blank control. The calibration curve was obtained with bacterial concentration as the *X*-axis and A/A_0_ as the *Y*-axis, with A referring to the reduction in OD450 nm from bacteria coating to the control and A_0_ to the reduction in OD450 nm from the highest concentration of bacteria to the control. The LOD was set as 3.3SD of the blank samples at OD450 nm.

#### 2.5.4. Cross-Reactivities of the Assay

Instead of *S*. Typhimurium, the other tested strains, including *S*. Indiana, *S*. Dublin, *S*. Agona, *S*. Paratyphi B CMCC 50094, *S*. Enteritidis ATCC 13076, *Vibrio parahaemolyticus* ATCC 17802, *E. coli* ATCC 12900, *Staphylococcus aureus* ATCC 25923 and *Pseudomonas aeruginosa* CMCC 10104, were heat-inactivated and adjusted to 10^8^ CFU/mL. The following procedures of VHH-AuNPs recognition and coloration were carried out as the previous descriptions, and the absorbance at 450 nm was measured by a microplate reader.

#### 2.5.5. Sample Preparation

Fresh lettuce was rinsed with distilled H_2_O and cut into about 1 cm^2^. After wiping them with 75% ethanol, the pieces of lettuce were irradiated with ultraviolet light for 20 min to inactivate the bacteria on their surface. Then, the bacteria were cultured for 12 h and plated to count the initial concentration. The lettuce samples were contaminated by adding 100 μL of *S*. Typhimurium, followed by transferring them to a sterilized tube by adding 900 μL of PBS buffer. After fully being ground and ultrasonicated, the supernatants were collected for heat inactivation for 20 min, which was further used for colorimetric analysis. 

## 3. Results

### 3.1. Synthesis, Optimization and Characterization of CD@AuNPs

CD@AuNPs were synthesized by a two-step reaction using cyclodextrin as a stabilizer and NaBH_4_ as a reducing agent. In mild conditions, upon mixing CD with gold precursors, NaBH_4_ reduction was performed to form the nanoparticles. The reaction was carried out and optimized on the types of CDs, pH conditions and the molar ratios of β-CD/HAuCl_4_ and NaBH_4_/HAuCl_4_. The synthesized nanoparticles were readily aggregated overnight and precipitated without protection from CDs at pH 6.0 and 9.0 or at n(β-CD):n(HAuCl_4_) = 1:1. Compared with unmodified α-CD and γ-CD, β-CD was much more efficient in controlling the particle size of AuNPs, partially contributed to the higher hydrophobicity of β-CD to be able to form stronger hydrophobic complexes with metallic format of gold to minimize the total system energy and prevent their coalescence or aggregation [23]. Then, the particle size of CD@AuNPs, one of the indicators of its catalytic activities, was evaluated by the shift in bandwidth, which had an inverse relationship with the size of nanoparticles [24]. Then, the peroxidase-like activities of fresh CD@AuNPs were compared at the same molar, and the intensities of particles in small diameters also helped to assess condition optimization (Figure S1). Thus, β-CD was employed for surface modification on AuNPs for further study, with the optimum conditions for β-CD@AuNPs as n(β-CD):n(HAuCl_4_) = 2:1 and n(NaBH_4_):n(HAuCl_4_) = 1:1.

**Figure 1:**
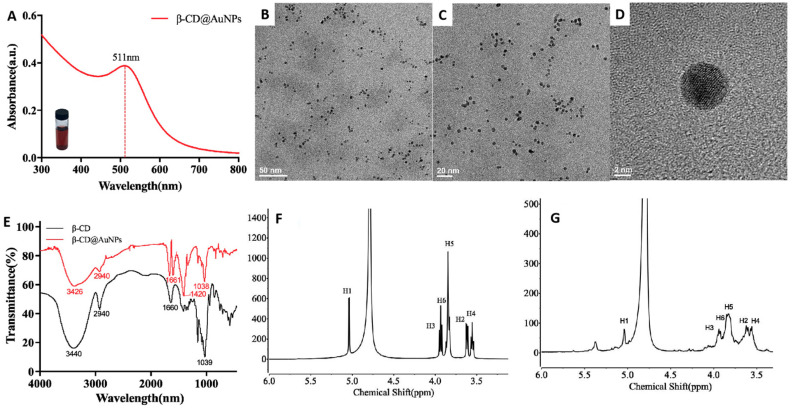
Synthesis and characterization of β-CD@AuNPs. Absorption spectra (**A**), TEM image (**B**–**D**), FTIR spectra (**E**), ^1^H NMR spectrum of β-CD (**F**) and β-CD@AuNPs (**G**).

The nanoparticles of β-CD@AuNPs exhibited a red color and revealed a strong plasmonic absorption at approximately 511 nm in UV-vis spectroscopy (Figure 1A). The expected spherical shape and homogeneity in an aqueous solution of β-CD@AuNPs was observed using TEM micrographs, which had their average sizes determined approximately to be 5 nm (Figure 1B–D). In order to verify the existence of β-CD capped on the surface of AuNPs, FTIR spectroscopy was analyzed with the same concentration of β-CD as control (Figure 1E). It was clearly observed that a new peak appeared at 1661 cm^−1^, which is the characteristic absorption of the carboxylic acid group (O-C-O/C=O) [15], revealing the surface modification of β-CD on AuNPs. The stretching vibration shifting from 3440 cm^−1^ to 3426 cm^−1^ and an obvious shrink of the peak areas at 1420 cm^−1^ and 1038 cm^−1^ implied the involvement of the O-H, C-H and C-O-H groups in the reduction of Au ions, but remained the skeleton structures of polysaccharides [25,26]. The results were further verified by analyzing the six chemical shifts in ^1^H NMR spectra (in D_2_O) of β-CD and β-CD@AuNPs (Figure 1F,G, Table S1). The lower signal and the broader peak of β-CD@AuNPs also implied that the β-CD was capped on the surface of gold nanoparticles. The results indicated the hydroxyl groups of β-CD were most likely to be oxidized to carboxyl groups, which provided sufficient strength to prevent agglomeration.

In the presence of β-CD, additional reducing agents, such as NaBH_4_, were capable of reducing the particle sizes during the reduction of HAuCl_4_ [27]. This might be due to NaBH_4_ at the initial stage of reducing Au (III) to Au (0), preventing the continued growth of AuNPs, which remained in small particle size. In addition, β-CD molecules can be directly employed as both reducing and stabilizing agents for β-CD@AuNPs preparation. However, upon the addition of β-CD to the HAuCl_4_ aqueous solution, the solution should be heated to 100 °C and lasted from 30 min to 24 h [15,16,18]. Although avoiding the excess reducing agents, the nanoparticles were formed in a diameter of ≈20 nm, which might illustrate some influences on their stabilities and catalytic activities.

### 3.2. Peroxidase-like Catalytic Activities of β-CD@AuNPs

**Figure 2:**
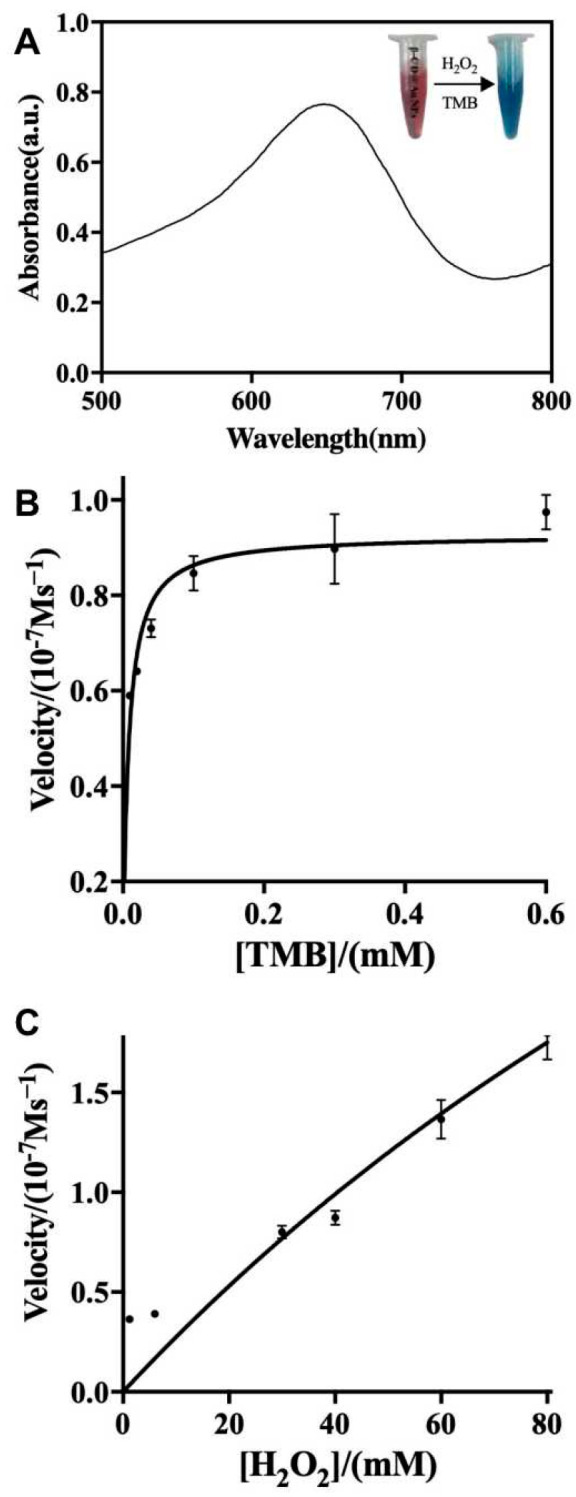
Peroxide-like catalytic properties of β-CD@AuNPs. (**A**) Absorption spectra under the optimized conditions; Michaelis–Menten kinetic assay of β-CD@AuNPs by varying substrate TMB (**B**) or H_2_O_2_ (**C**).

Peroxidase-like activities of β-CD@AuNPs were investigated and verified in catalysis of peroxidase substrates 3,3′,5,5′-tetramethylbenzidine (TMB) to oxidation state oxTMB, which had a blue color change in the presence of H_2_O_2_ and presented a maximum absorption at 652 nm (Figure 2A). The β-CD capped on the surface of AuNPs could significantly enhance the peroxidase-like behaviors, which were strongly dependent on pH conditions, temperature, incubation time and substrate concentrations (Figure S2). Appropriate OD values were obtained in a weakly acidic citrate buffer (pH 3.5) for an incubation of 30 min to avoid the speedy reactions at pH 3.0. Then, the peroxidase-like performances of TMB oxidization by β-CD@AuNPs were described by the responses of varying H_2_O_2_ and TMB, respectively [28]. Thus, the optimized conditions for the peroxidase-like performances of β-CD@AuNPs were obtained with 15 µL of H_2_O_2_ (30%) and 25 µL of TMB (5 mM) prepared in 0.05 M citrate buffer (pH 3.5) at 37 °C for 10 min. The kinetic parameters, which related reaction rates to substrate concentrations, were calculated to be consistent with the typical Michaelis–Menten curve to support the peroxidase-like behavior of β-CD@AuNPs toward both substrates, H_2_O_2_ and TMB (Figure 2B,C). The Km values for β-CD@AuNPs toward TMB were much lower than those toward H_2_O_2_, indicating that β-CD@AuNPs possessed a stronger affinity toward TMB with a fixed H_2_O_2_ concentration (Table S2).

Currently, natural peroxidase enzymes are generated from microorganisms or plants for a series of industrial applications. However, their catalytic activities were sensitive to environmental conditions and generally deactivated under variable pH and temperature conditions [29]. In this study, β-CD@AuNPs possessed stable properties and exhibited high peroxidase-like activities under a wide range of temperatures (25–75 °C), while their catalytic activities were lost when β-CD@AuNPs were incubated at 85 °C (or above) for 10 min (Figure S3A). Similarly, the peroxidase-like activities were significantly influenced by ionic strengths (Figure S3B), which might be attributed to the spoilage of surface interactions among the nanoparticles. It is worthwhile to mention that the presence of adamantane represented an inhibition of the peroxidase-like activities of β-CD@AuNPs (Figure S3C), which might be caused by occupying the inner cavity of β-CD to form a host–guest inclusion complex, thus leading to the changes in structures and functionalities. These results indicated that the surface modification of β-CD on Au nanoparticles enhanced its peroxidase-like activities of bare Au, and their synergistic effects of both β-CD and AuNPs determined the high catalytic activities of β-CD@AuNPs.

### 3.3. Colorimetric Determination for Ascorbic Acid in ddH_2_O

**Figure 3:**
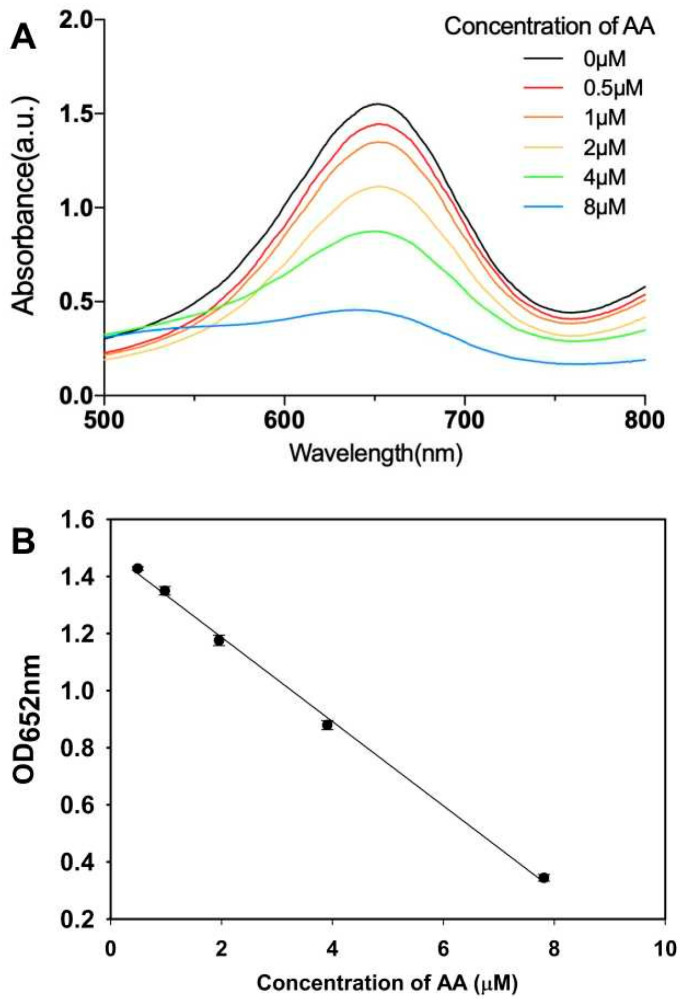
Absorption spectra (**A**) and calibration curve (**B**) of colorimetric determination for ascorbic acid based on β-CD@AuNPs.

Ascorbic acid (AA), or vitamin C, is an essential nutrient for human beings, which can only be absorbed from external sources, such as vegetables and fruits. In the presence of β-CD@AuNPs, the oxidation of TMB was ascribed to the electron transfer of reactive oxygen species production by H_2_O_2_ [30]. As AA molecules are antioxidants, they exhibit great reducibility of oxTMB to TMB, resulting in the blue color fading to colorless. Herein, the inhibition of peroxidase-like activities of β-CD@AuNPs can be used to develop the quantitative colorimetric detection of AA. The oxTMB was first produced by TMB oxidization in the catalysis of β-CD@AuNPs, and then a series of concentrations of AA were added to reduce oxTMB to TMB. The absorbance at 652 nm decreased with increasing concentrations of AA (Figure 3A). The linear calibration curve between the absorbance of oxidized TMB at 652 nm and the concentration of AA was acquired, indicating a quantitative good relationship ranging from 0.5 μM to 8.0 μM (R^2^ = 0.999). The limit of detection for AA was calculated to be 0.2 μM (Figure 3B), which exhibited comparable sensitivities with the previous sensors based on peroxidase-like activities of Au nanoparticles [31]. To evaluate the selectivity of this assay for AA molecules, higher concentrations of ions, amino acids and carbohydrates were used to reduce oxTMB. The results displayed that except 200 μM of L-cystine and BSA, no obvious inhibition was observed with a much higher concentration of interfering substances, including Na^+^, K^+^, Ca^2+^, glucose, maltose, sucrose, fructose, histidine, valine and glycine (Figure S4). Therefore, the colorimetric detection based on β-CD@AuNPs in this study showed good selectivity for the determination of AA molecules.

### 3.4. Development of Colorimetric Detection for S. Typhimurium

Enzyme-linked immunosorbent assay (ELISA) is a well-established colorimetric detection technique for monitoring *Salmonella* contamination in the food industry. The detection signals of color change were produced by TMB oxidization in the catalysis of peroxidases. Theoretically, nanozyme, which performs peroxidase-like activities, can be used in ELISA detection for analytical and therapeutic applications. However, the conjugation of nanozymes with biological probes, such as specific antibodies or aptamers, is still a challenge, where convenient methods without compromising catalytic activities are needed [32]. In this study, ada worked as a guest molecule, can bind the cavity of β-CD in a stable host–guest interaction [33], which was used as a “coupling bridge” to conjugate gold nanoparticles and antibodies to construct a bifunctional probe, simultaneously processing both antigen-specific binding abilities and peroxidase-like activities of β-CD@AuNPs.

**Figure 4:**
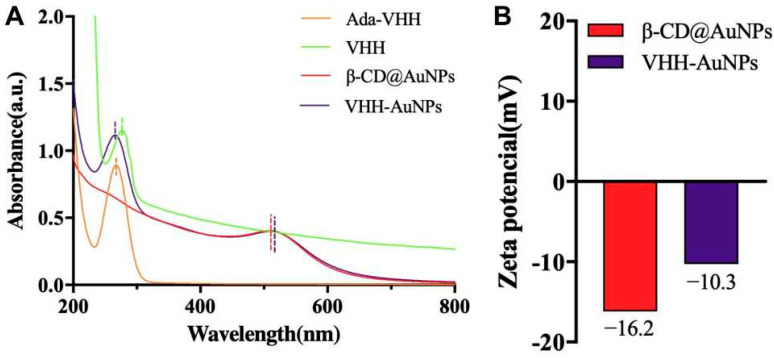
Construction of VHH-AuNPs through host–guest interactions and confirmed by UV-Vis absorption spectra (**A**) and zeta potential analysis (**B**).

Herein, nanobodies, also referred to as VHHs, were the variable heavy-chain domains derived from immunoglobulin heavy-chain antibodies in Camelidae [34] or sharks [35]. Owing to the monovalent properties, VHHs were the smallest but complete antigen-binding units and can bind at a higher density on the surface of antigens to increase the signal-to-noise ratio, resulting in enhancing the assay sensitivities. VHH-9-28 against *S*. Typhimurium was expressed and confirmed by SDS-PAGE analysis and UV-vis spectra (Figure S5). The conjugation of ada-VHH was constructed and confirmed by the shifting of the absorption peak from 280 nm of VHH to 268 nm (Figure 4A). Then, the VHH-AuNPs were successfully synthesized through the host–guest interaction between β-CD and ada, which was further verified by the absorption peak both at 266 nm and 514 nm and the surface zeta potential changing to −10.3 mV from −16.2 mV of β-CD@AuNPs (Figure 4B). However, compared with bare β-CD@AuNPs, the peroxidase-like activities of VHH-AuNPs were analyzed to show a half reduction because of the occupation of ada on the inner cavity of β-CD (Figure S3).

**Figure 5:**
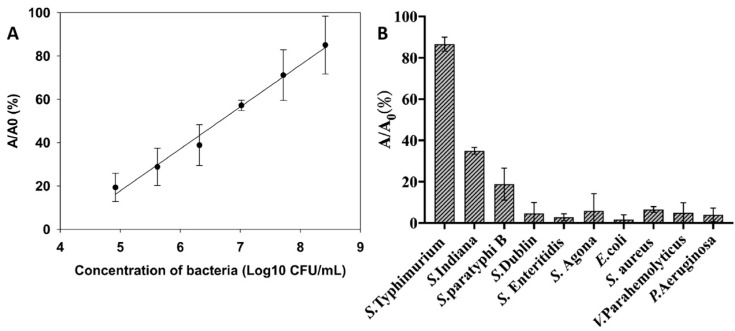
Establishment of the calibration curve for the detection of *S*. Typhimurium (**A**) and cross-reactivities with other *Salmonella* serotypes and non-*Salmonella* strains (**B**) based on the antigen-binding capacities and peroxidase-like activities of VHH-AuNPs. Errors bars represent the standard deviation of three replicates.

The one-step ELISA for *S*. Typhimurium was developed by the incubation of a series of concentrations of antigens and, afterward, the addition of VHH-AuNPs. The linear calibration curve was established with VHH-AuNPs acting both as the capture and detecting probe, indicating a good linear correlation between the bacterial concentration and A/A_0_ in the range of 8.3 × 10^4^ CFU/mL~2.6 × 10^8^ CFU/mL (Figure 5A), with a limit of detection of 2.5 × 10^4^ CFU/mL. The cross-reactivity analysis implied that the one-step ELISA showed binding capacities with the target strain *S*. Typhimurium ATCC14028 and cross-reactivities with *S*. Indiana and *S*. Paratyphi B, which was consistent with the specificities of VHH (data not published). There was no significant response for the remaining *Salmonella* serotypes and four non-*Salmonella* strains (Figure 5B).

To verify its application in food samples, the established one-step ELISA was employed to detect the various concentrations of *S*. Typhimurium spiked in sterilized lettuce samples prepared by UV irradiation. The initial bacterial concentration (3.06 × 10^8^ CFU/mL) was determined by counting colonies on plates and then diluted 10-fold to add to the lettuce samples. The bacteria were detected using one-step ELISA and verified using culture-based methods. The one-step ELISA was consistent with the results of culture-based methods (Figure S6) but was shown to be less time-consuming. The recoveries, ranging from 97.31% to 103.29%, revealed that the established VHH-AuNPs-based one-step ELISA was feasible for detecting *S*. Typhimurium in lettuce (Table 1).

Utilizing the supramolecular assembly aggregation induced by hexadecyl trimethyl ammonium bromide (CTAB), the rapid detection for *S*. Typhimurium can be developed based on β-CD@AuNPs [17] but lacks of the targeting recognition. Therefore, in order to develop a colorimetric assay for food pathogens, specific antibodies, one of the preferred candidates for antigen binding, should be bioconjugated or physisorpted with gold nanoparticles [36]. Ideally, the conjugation step should preserve both the catalytic activities of nanozymes and the recognition capacities of antibodies. However, regardless of glutaraldehyde crosslinking and the streptavidin–biotin system, which were the commonly used choices for covalent conjugation or adsorption, the catalytic activities of nanozymes might be inhibited by blocking their surfaces and hindering their actions on substrates [37]. There were rare exceptions that positively charged gold nanoparticles (+AuNPs) conjugated with anti-HA antibodies through electrostatic interactions maintained the peroxidase-like activities in colorimetric analysis for influenza virus [38]. At the same time, in this study, β-CD-capped AuNPs were synthesized, performing excellent peroxidase-like activities, then incubated with VHHs through the host–guest interactions to form a strong and stable conjugation, allowing for antigen-specific recognition and signal amplification. Although β-CD@AuNPs conjugating with VHH led to the half-decreasing signal at OD652 nm, no significant influences on the binding capacities of VHHs toward *S*. Typhimurium were observed, and acceptable peroxidase-like activities of β-CD@AuNPs were maintained. To our knowledge, β-CD@AuNPs were the first attempt to bind with VHHs through the host–guest interactions, integrating both catalytic activities catalysis and antigen-binding activities to develop one-step colorimetric detection for *S*. Typhimurium.

## 4. Conclusions

In order to enhance the peroxidase-like activities of AuNPs for application in colorimetric detection in the food industry, fine-tuning their size and surface modification was explored. β-CD@AuNPs were synthesized by NaBH_4_ reduction and stabilized by β-CD under alkaline conditions. The excellent peroxidase-like behaviors of β-CD@AuNPs were demonstrated and confirmed to be consistent with the typical Michaelis–Menten curve toward both substrates, H_2_O_2_ and TMB. By taking advantage of this property, a colorimetric detection for ascorbic acid in ddH_2_O was established. Meanwhile, a bifunctional probe VHH-AuNPs, covalent by the unique host–guest interactions between β-CD and ada, was further employed to develop one-step colorimetric detection for *S*. Typhimurium and was shown to be effective when applied to spiked lettuce samples. Therefore, because of the enhanced peroxidase-like activities, the method based on β-CD@AuNPs in this study may represent an attractive approach for developing colorimetric sensing platforms for food quality and safety monitoring.

## Figures and Tables

**Table 1:** Recovery of *S*. Typhimurium spiked in lettuce samples (*n* = 3).

Spiked Concentration (CFU/mL)	Detected Concentration (Average, CFU/mL)	Recovery (%)
3.06 × 10^8^	(3.16 ± 0.10) × 10^8^	103.29 ± 3.26
3.06 × 10^7^	(3.14 ± 0.09) × 10^7^	102.69 ± 2.94
3.06 × 10^6^	(2.98 ± 0.14) × 10^6^	97.31 ± 4.57

## Data Availability

The data presented in this study are available upon request from corresponding author.

## Supplementary Materials

The following supporting information can be downloaded at: https://www.mdpi.com/article/10.3390/bios14040169/s1, Figure S1: Optimization of CD@AuNPs synthesis; Figure S2: Optimization of β-CD@AuNPs on peroxidase-like activity performances; Figure S3: Influences on the peroxidase-like performances of β-CD@AuNPs; Figure S4: Selectivities of colorimetric detection of AA; Figure S5: Expression and purification of VHH. Figure S6: Correlations between methods; Table S1: ^1^H NMR Chemical shifts of β-CD and β-CD@AuNPs; Table S2: Kinetic parameters of peroxidase-like activity of β-CD@AuNPs.
