# Supplementary material for: Synthesis of β-Cyclodextrin@gold Nanoparticles and Its Application on Colorimetric Assays for Ascorbic Acid and Salmonella Based on Peroxidase-like Activities
Source: Biosensors (Basel). 2024 Mar 31;14(4):169. doi: 10.3390/bios14040169 (PMC11048340; doi:10.3390/bios14040169)
Supplement: Supplementary file 1 [file biosensors-14-00169-s001.zip › biosensors-2935640-supplementary.pdf]

Supplementary file for

Synthesis of β-Cyclodextrin@gold Nanoparticles and Its Application on Colorimetric Assays for Ascorbic Acid and *Salmonella* Based on Peroxidase-like Activities

Xinyi Fan [1,†], Yuexin Bao [1,†], Yanhong Chen [1], Xiaohong Wang [1,2], Stephen L. W. On [3] and Jia Wang [1,2,*]

[1] College of Food Science and Technology, Huazhong Agricultural University, Wuhan 430070, China

[2] Key Laboratory of Environment Correlative Dietology, Huazhong Agricultural University, Wuhan 430070, China

[3] Department of Wine, Food and Molecular Biosciences, Faculty of Agriculture and Life Sciences, Lincoln University, Lincoln 7647, New Zealand

* Correspondence: wangjia@mail.hzau.edu.cn; Tel.: +86-27-87282111

† These authors contributed equally to this work.

Table of content

Figure S1. **Optimization of CD@AuNPs synthesis. Absorption spectra and the** peroxidase-like activities **of fresh CD@AuNPs under the conditions of types of CD (A, B), pH (C, D), the molar ratio of CD:$HAuCl_4$ (E, F). The Absorption spectra and particle size of $NaBH_4$:$HAuCl_4$ (G, H)....**...3

Figure S2. Optimization of β-CD@AuNPs on peroxidase-like activity performances. (A) pH; (B) incubation temperature; (C) incubation time; (D) concentration of H_2O_2; (E) concentration of TMB..4

Figure S3. Influences of temperature (A), ionic strength (B) and concentration of Ada (C) on the peroxidase-like performances of β-CD@AuNPs...5

Figure S4. Selectivities of colorimetric detection of AA based on β-CD@AuNPs.......6

Figure S5. Expression and purification of VHH confirmed by SDS-PAGE analysis (A) and UV-Vis absorption spectra (B)...7

Figure S6. Correlations between one-step ELISA and culture-based methods in spiked lettuce..8

Table S1. ^{1}H NMR Chemical shifts corresponding of β-CD and β-CD@AuNPs.........9

Table S2. The kinetic parameters of peroxidase-like activity of β-CD@AuNPs........10

Figure S1. Optimization of CD@AuNPs synthesis. Absorption spectra and the peroxidase-like activities **of fresh CD@AuNPs under the conditions of types of CD (A, B), pH (C, D), the molar ratio of CD:$HAuCl_4$ (E, F). The Absorption spectra and particle size of $NaBH_4$:$HAuCl_4$ (G, H).**

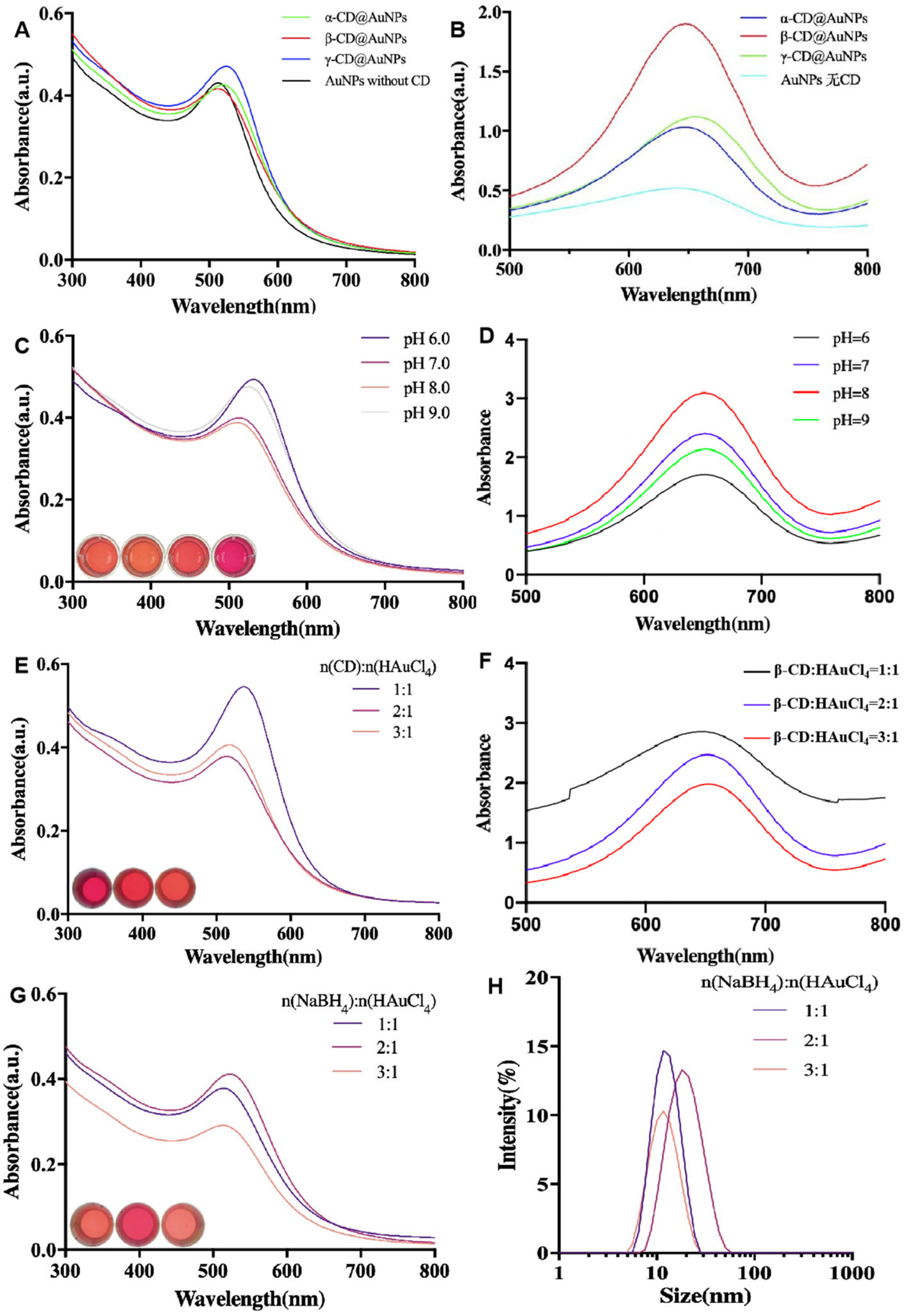

Figure S2. Optimization of **β-CD@AuNPs on peroxidase-like activity performances. (A) pH; (B) incubation temperature; (C) incubation time; (D) concentration of H_2O_2; (E) concentration of TMB.**

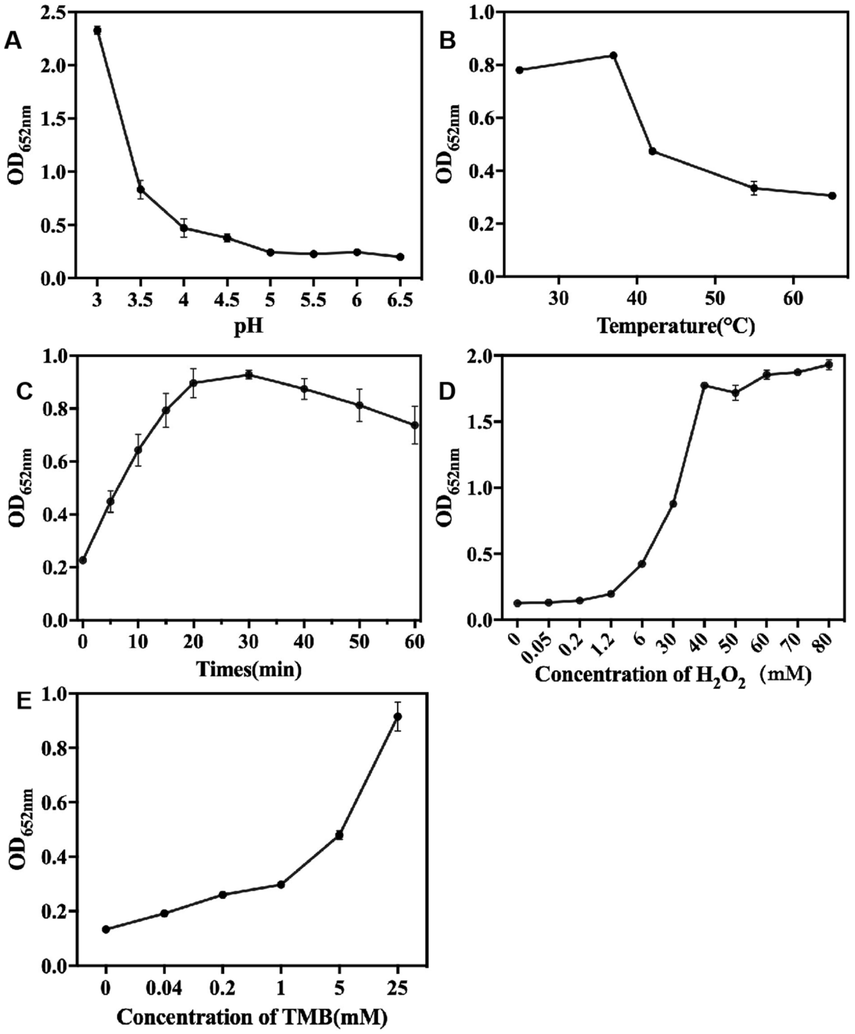

Figure S3. Influences of temperature (A), ionic strength (B) and concentration of Ada (C) on the **peroxidase-like performances of β-CD@AuNPs.**

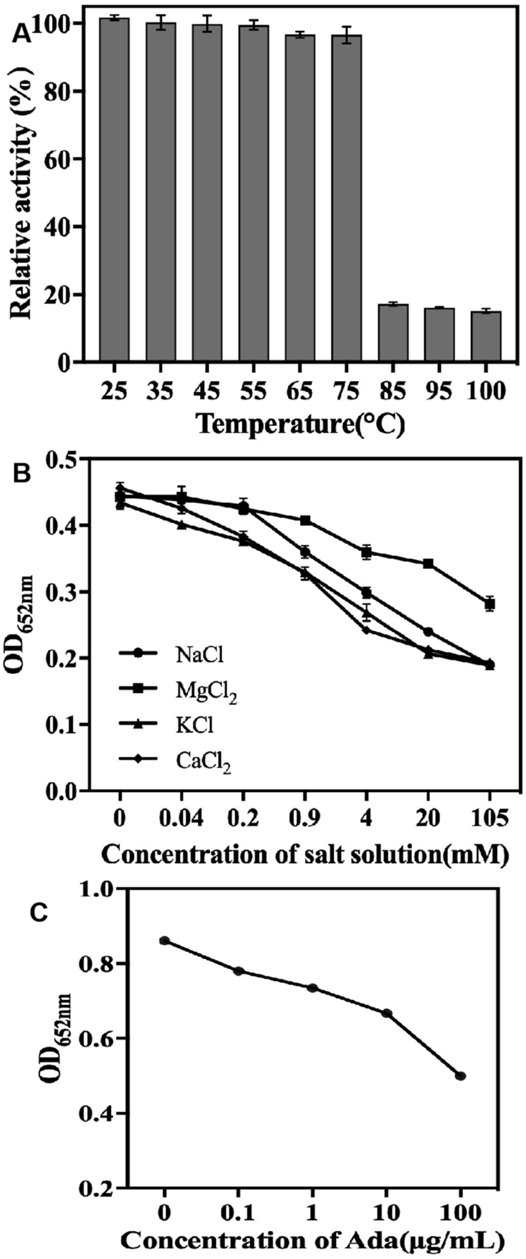

Figure S4. Selectivities of colorimetric detection of AA based on β-CD@AuNPs

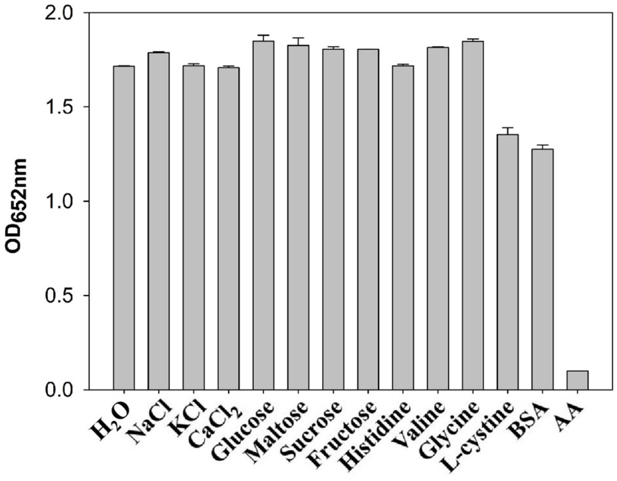

Figure S5. Expression and purification of VHH confirmed by SDS-PAGE analysis (A) and UV-Vis absorption spectra (B).

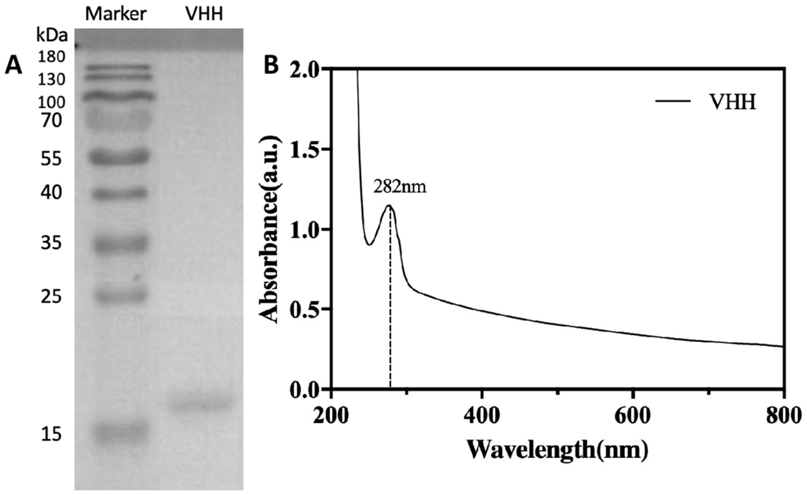

Figure S6. Correlations between one-step ELISA and culture-based methods in spiked lettuce.

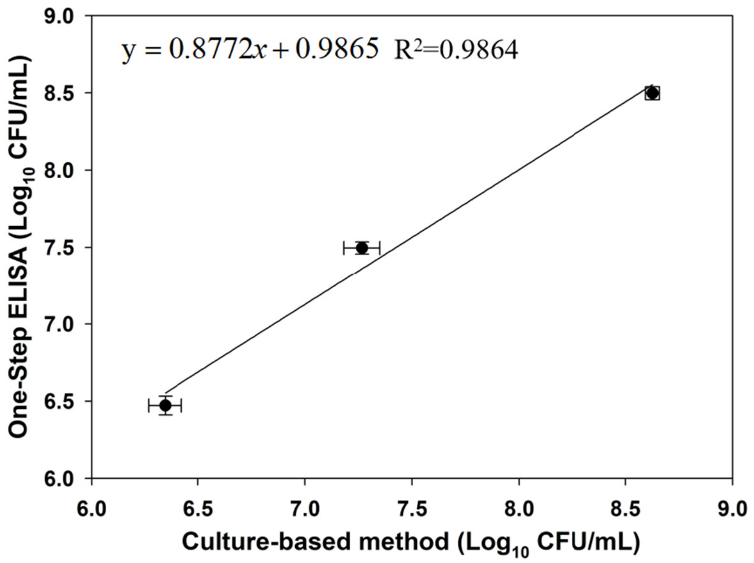

Table S1. ^{1}H NMR Chemical shifts corresponding of β-CD and β-CD@AuNPs.

	H1	H2	H3	H4	H5	H6
β-CD	5.0353	3.6228	3.9517	3.5538	3.8456	3.9355
β-CD@AuNPs	5.0398	3.6201	3.9382	3.5574	3.8236	3.9224

Table S2. The kinetic parameters of peroxidase-like activity of β-CD@AuNPs.

Catalyst	Substrate	V_{max}/M•s^{-1}	K_m/(mM)
β-CD@AuNPs	TMB	0.927×10^{-7}	0.0074
β-CD@AuNPs	H_2O_2	7.517×10^{-7}	263.5
